# Arbuscular mycorrhizal fungi in oat-pea intercropping

**DOI:** 10.1038/s41598-022-22743-7

**Published:** 2023-01-09

**Authors:** Alan Lee, Patrick Neuberger, Akim Omokanye, Guillermo Hernandez-Ramirez, Keunbae Kim, Monika A. Gorzelak

**Affiliations:** 1 Peace Country Beef and Forage Association, Fairview, AB T0H1L0 Canada; 2 Agriculture and Agri-Food Canada, Lethbridge Development and Research Centre, 5403-1 Avenue South, Lethbridge, AB T1J 4B1 Canada; 3 Department of Renewable Resources, University of Alberta, Edmonton, AB T6G 2R3 Canada; 4 Katholieke Universiteit Leuven, Oude Markt 13, 3000 Leuven, Belgium

**Keywords:** Ecology, Microbiology, Plant sciences, Environmental sciences

## Abstract

Arbuscular mycorrhizal fungal diversity can be altered by intercropping plant species, as well as N fertilizer applications. This study examined the effects of oat-pea intercropping and N fertilizer addition on the richness and diversity of mycorrhizal species, as well as identified the most common arbuscular mycorrhizal fungi (AMF) genera recruited for oats and peas in two growing seasons (2019 and 2020). The AMF diversity was higher in an intercropped system compared to their respective monocropping system. Under drier conditions in 2019, arbuscular mycorrhizal richness decreased with N fertilizer addition in sole peas and increased with N fertilizer addition in sole oats, but no significant change in richness was observed in oat-pea intercropping. During the wetter growing season 2020, arbuscular mycorrhizal diversity increased when oat and pea were intercropped, compared to either sole oat or sole pea. *Diversispora* in sole pea was a significant indicator differentiating the root associated AMF community from sole oat. *Claroideoglomus* richness increased in peas in 2020, thus this genus could be moisture dependent. *Paraglomus* richness in oat-pea intercropping was similar to sole oat in 2019, and similar to sole pea in 2020. This can suggest that *Paraglomus* is an indicator of plant stress under intercropping, as based on the premise that stressed plants release more exudates, and the subsequent mycorrhizal associations favor these plants with higher exudation. Future investigations can further reveal the functions and benefits of these mycorrhizal genera in annual monocrop and intercropping systems.

## Introduction

Intercropping can provide many benefits. Certain intercropping options have demonstrated overyielding capacity^1,2^ or yield stability under contrasting environments^3–5^. Furthermore, intercropping forage systems have delivered consistently better nutritive quality^3^.

Benefits of intercropping include the capacity to reduce N fertilizer input by increasing N use-efficiency. This is in part because N loss to the environment is a major pollution source through N leaching, runoff, or emissions^6–8^. Preventing N overfertilization and optimizing N fertilizer usage can be pursued by accounting for the underlying soil moisture availability^9^ as well as by choosing an appropriate N fertilizer rates^10^. Additionally, N fertilizer rates need to account for any interaction effects between plant species within a given intercropping system (e.g., contribution of legume crops to overall N availability).

While most of the effects of intercropping are attributed to interactions between plant species, there are studies demonstrating that certain plant-microbial interactions are also involved; specifically the interaction between plants and soil mycorrhizal fungi^11,12^. There is also evidence from multi-species fields that plants can share nutritional resources via transport channels created by mycorrhizal symbiosis^13,14^. Where plants acquire or exchange more nutrients through these mycorrhizal channels, they could require less exogenous fertilizers.

There are many studies that focus on the role of arbuscular mycorrhizal fungi (AMF) between perennial crops growing together^15–18^, and fewer on annual crop species^19,20^, and seldom in intercropping systems^13^. Relative to oat and pea monocrops, this study examines how oat-pea intercropping with and without N fertilizer addition affects AMF richness and diversity, including both the bulk soil and root compartments. In line with previous studies on perennial plant species, we anticipate that the AMF root community to change as a response to intercropping and N fertilizer addition.

## Methods

### Site description and experimental design

Field experiments were carried out over two growing seasons—from 30 May to 30 Aug. 2019, and from 22 Jun. to 21 Sep. 2020, at the Fairview Research Farm, located in north-western Alberta, Canada (56° 04′ 53.3″ N, 118° 26′ 25.1″ W). The study was conducted using new plots each year. This region experiences long and cold winters, short and mild summers, and is characterized as a boreal climate. The soil at the experimental site is classified as an Eluviated Black Chernozem, according to the Canadian System of Soil Classification^21^. The management history of the experimental sites was a long-term alfalfa stand for hay production until termination with Roundup WeatherMax^®^ herbicide (glyphosate) the fall of 2018 (September) before the experiments commenced^3^. The long-term temperature and rainfall averages, as well as the observed temperature and rainfall for 2019 and 2020 were acquired through the Alberta Climate Information System (ACIS 2020) from a permanent weather station located on site (Table 1). Baseline soil sample collection and analyses were done before crop seeding in both years. Soil availabilities of nitrogen (N) and phosphorus (P) were deficient. Upon alfalfa stand termination in fall 2018, the field was deep plowed, disced and harrowed. The sites were lightly harrowed before seeding operations.

**Table 1:** Rainfall, temperature, and soil properties (0–15 cm depth increment) at the experimental site, as well as the basal fertilizer applied in 2019 and 2020.

	2019	2020	Long term average
Month	Rainfall (mm)		
June	72.9	67.2	64.5
July	61.9	89.8	69.5
August	49.1	53.9	47.5
September	24.6	23.1	33.7
**Air temperature (°C)**			
June	14.1	14.0	14.0
July	15.1	15.6	15.9
August	12.9	14.1	14.6
September	9.7	10.3	9.6
**Soil property**			
Nitrate–N (mg kg^−1^)	5	24	
P (mg kg^−1^)	7	10	
K (mg kg^−1^)	242	216	
S (mg kg^−1^)	5	5	
Ca (mg kg^−1^)	1920	1760	
Mg (mg kg^−1^)	354	359	
pH	6.2	6.2	
Organic matter, OM (%)	8.2	3.7	
**Fertilizer blend applied**			
P_2_O_5_ (kg ha^−1^)	27.2	10.9	
K_2_O (kg ha^−1^)	27.2	Nil^a^	
S (kg ha^−1^)	12.7	13.6	

^a^Soil tests indicated adequate availability of soil K, so no additional K_2_O was applied.

The two plant species in this experiment were oats (*Avena sativa* L.*,* O), peas (*Pisum sativum* L.*,* P), and were intercropped as oat-pea (OP). Seeding rates had the plant density targets of 300 plants m^−2^ for monocrop oats and 90 plants m^−2^ for monocrop peas. Within the 2-species intercropping, each plant species was seeded at 75% of their common seeding rates.

A factorial randomized complete block design was implemented, where the two factors were intercropping systems (O, P, OP) and with or without N fertilizer (fertilizer rate was applied at 75 kg N ha^−1^) with four replicates. On 30 May, 2019 and 22 Jun., 2020, we used a 6-row small-plot seeder (equipped with disc-type openers) with a 22.9 cm row spacing to seed into 2 m by 16 m plots, and the base fertilizer (a mixture of P, K, and S, Table 1) and N fertilizer addition (granular urea, 46-0-0) were banded at seeding. To reduce weed competition, we applied glyphosate (StartUp^®^ Roundup) herbicide as pre-emergent in 2019 and pre-pass in 2020 as well as occasional hand weeding.

### Field sample collection

For mycorrhizal analytical samples, three locations within each experimental plot were sampled using a 70% ethanol cleaned trenching shovel, and individually separated into three bulk soil samples and three plant root samples. The plant root samples were washed with distilled water in trays to remove adhering soils and clean roots were then subsampled for microscopy and molecular analysis, where the molecular samples were stored at – 20 °C while the microscopy samples were stored in ethanol until ready for analysis. Bulk soil samples were not subsampled and were also stored at – 20 °C until ready for analysis.

Biomass was harvested to determine forage yield (biomass productivity) in addition to plant water use-efficiency (WUE) and forage nutritional quality to address research questions intercropping productivity as an alternative forage source to conventional cattle feed^3^.

### Root colonization

Root samples were washed with distilled water until free of soil. 0.1 g of root sample is collected and stained using the procedure detailed in McGonigle^22^, where roots were cleared in 10% KOH and stained with Trypan blue solution for 30 min, stored on slides at 4 °C until examined. The roots were then evaluated on a compound microscope at 40× magnification and counted for hyphae, arbuscules, and vesicles per root intersection. Each part is recorded as a ratio of hyphae/arbuscule/vesicle count: intersection.

### Molecular analysis

Total DNA was extracted from 0.25 g of samples using the PowerSoil DNA Isolation kit according to the manufacturers’ guideline (MoBio Laboratories Inc., Carlsbad, California). DNA purity and concentration were measured by a Biodrop spectrophotometer (Biochrom, Cambridge, UK) and a Qubitv4 fluorimeter using a Qubit™ dsDNA BR Assay Kit (ThermoFisher Scientific, Massachusetts, USA), respectively. The amplification capability of the AMF small subunit (SSU) region were confirmed with a polymerase chain reaction (PCR) test by using primer pair NS31 (5′-TTGGAGGGCAAGTCTGGTGCC-3′) to AML2 (5′-GAACCCAAACACTTTGGTTTCC-3′). The PCR cycling conditions were: 94 °C (3 min); 35 cycles of 94 °C (45 s), 63 °C (60 s), and 72 °C (90 s); followed by final extension step 72 °C (10 min) (Morgan et al.)^23^. The fragment size and quality of amplification of the PCR product was verified by electrophoresis on 1% agarose gel.

Sequencing was implemented using an Illumina MiSeq PE300 platform at a read length of 2 × 300 bp (Illumina Inc., San Diego, California, USA). Using the same primer pair as the set used in PCR test, the metagenomics data was extracted from the samples. The metagenomics data was then processed further using the Fluidigm sequencing adaptor to produce a genetic library. The library prep was completed by Genome Quebec (Quebec, Canada).

### Bioinformatics and statistical analysis

The raw FASTQ data was processed with the Qiime2 pipeline (version 2019.10 https://qiime2.org/)^24^. DADA2 algorithm was used to implement error correction, quality filtering, chimera removal and sequence variance of Illumina amplicon sequences^25^. The first 21 bp and 22 bp in the forward and reverse reads were trimmed for removing primers, respectively. The forward and reverse reads were truncated at 295 and 283 bp, corresponding to average quality score (Phred Q score) of higher than 20, respectively. These quality criteria encompassing denoised, merged, and non-chimeric yields a loss of 73.5% to whole sequence reads. Operational taxonomic units (OTUs) were clustered with ≥ 97% similarity in an open-reference picking process using classify-consensus-vsearch in Qiime2. Clustered OTUs were used to query the MaarjAM database^26^. Unassigned OTUs were further aligned against the Silva SSU 138 database at 99% sequence similarity. Non-Glomeromycotina fungi for the 18S sequences were removed from subsequent analyses. Singletons and OTUs present in less than three samples were removed from the analysis. The processed data were exported from Qiime2 to analyze and visualize within the R packages ‘phyloseq’^27^ and ‘vegan’^28^.

Alpha diversity of AMF communities was evaluated by Chao1 richness, Pielou’s evenness, Shannon’s diversity, and inverse Simpson’s diversity indices with a linear mixed model as the parametric test. The linear mixed model was used for analysis of variance (ANOVA) of AMF colonization as well as ANOVA of alpha diversity. By doing so, a random effect (‘block’) was removed while statistically analyzing crop and fertilizer effect on colonization and alpha diversity using the R package. Normality and homoscedasticity of the model residuals were assessed using Shapiro–Wilk and Levene test function, respectively^29^. Box-Cox transformation were applied to correct non-normality or heteroscedasticity when needed. Pairwise comparisons were conducted after a significant ANOVA with Tukey’s Honest Significant Difference (HSD) using agricolae package in R^30^.

Before statistical analysis of beta diversity, OTUs absolute count data were transformed for even sampling depth based on ‘phyloseq’ tutorial^31^. Beta diversity of AMF communities was assessed by permutational multivariate analysis of variance (PERMANOVA) and illustrated by non-metric multidimensional scaling (NMDS) using Bray–Curtis dissimilarity matrices^32^. The differential abundance test was performed by edgeR: a Bioconductor package^33^. Transformation-based canonical correspondence analysis (tb-CCA) and redundancy analysis (tb-RDA) were used to explain dissimilarity with environmental variables. Taxa were agglomerated at the genus level using phyloseq^31^ for correlations to physicochemical characteristics. Spearman correlation test was performed to find relationships between dominant genera and environmental variables.

### Statement on plant material collected

Plant material was collected with permission on the Peace Country Beef and Forage Association experimental farm in Fairview, Alberta. Collection complied with all institutional, and national guidelines and legislation.

## Results

### Biomass yield and WUE

Biomass increased with precipitation, as expected and reported in Lee^3^. There was less water uptake in intercropping compared to sole oat and pea. In general, intercropping represented the median of the two sole cropping treatments, where oat had the highest biomass and WUE while pea had the lowest, and where pea had the highest mineral content and oat had the lowest^3^. Intercropping resulted in advantages in forage yield stability and was not associated with changes to the AMF community.

### Alpha diversity

**Figure 1:**
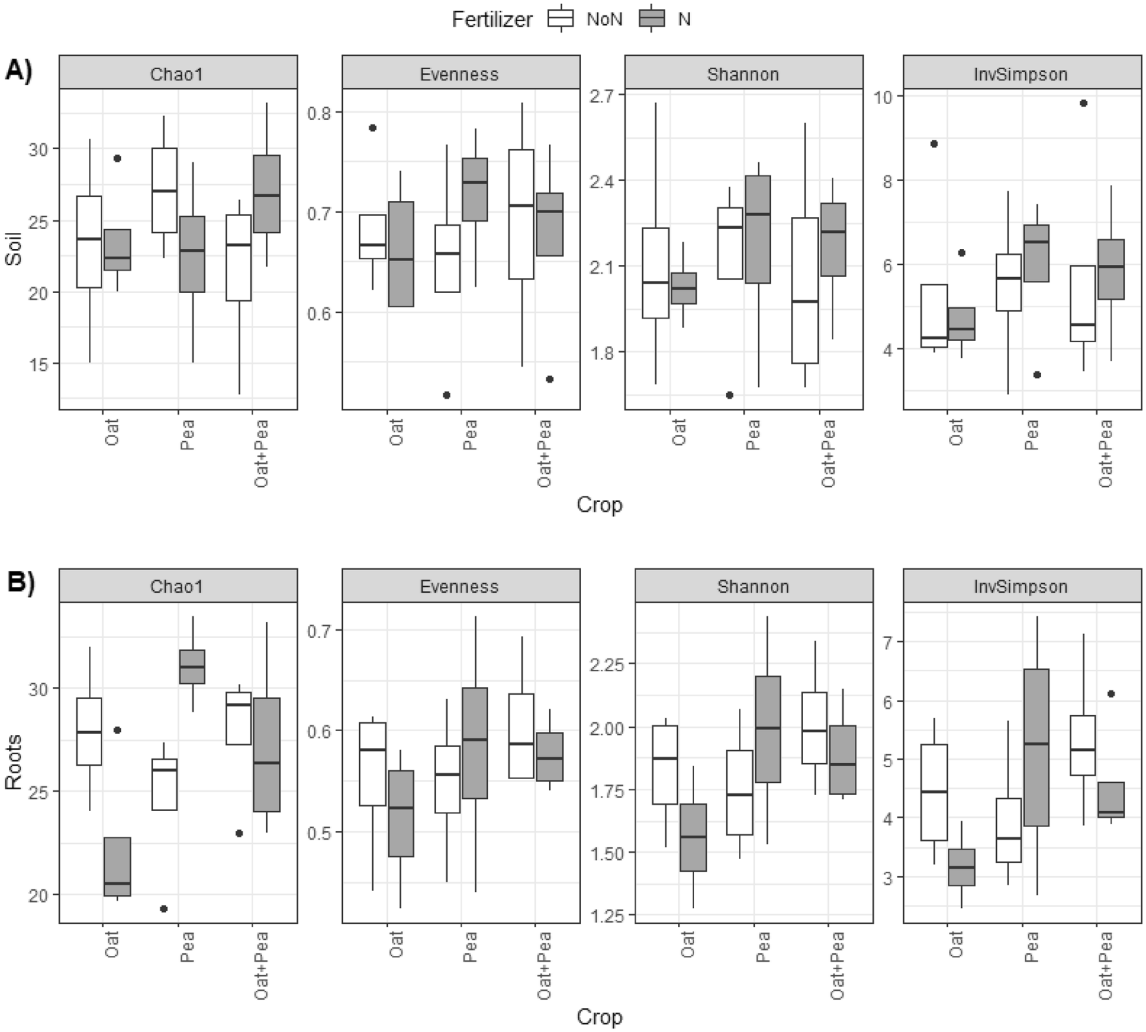
Arbuscular mycorrhizal fungi (AMF) alpha diversity including Chao1 richness, Peilou’s Evenness, Shannon diversity and inverse Simpson diversity across cropping systems at (**A**) bulk soil and (**B**) roots in 2019.

**Figure 2:**
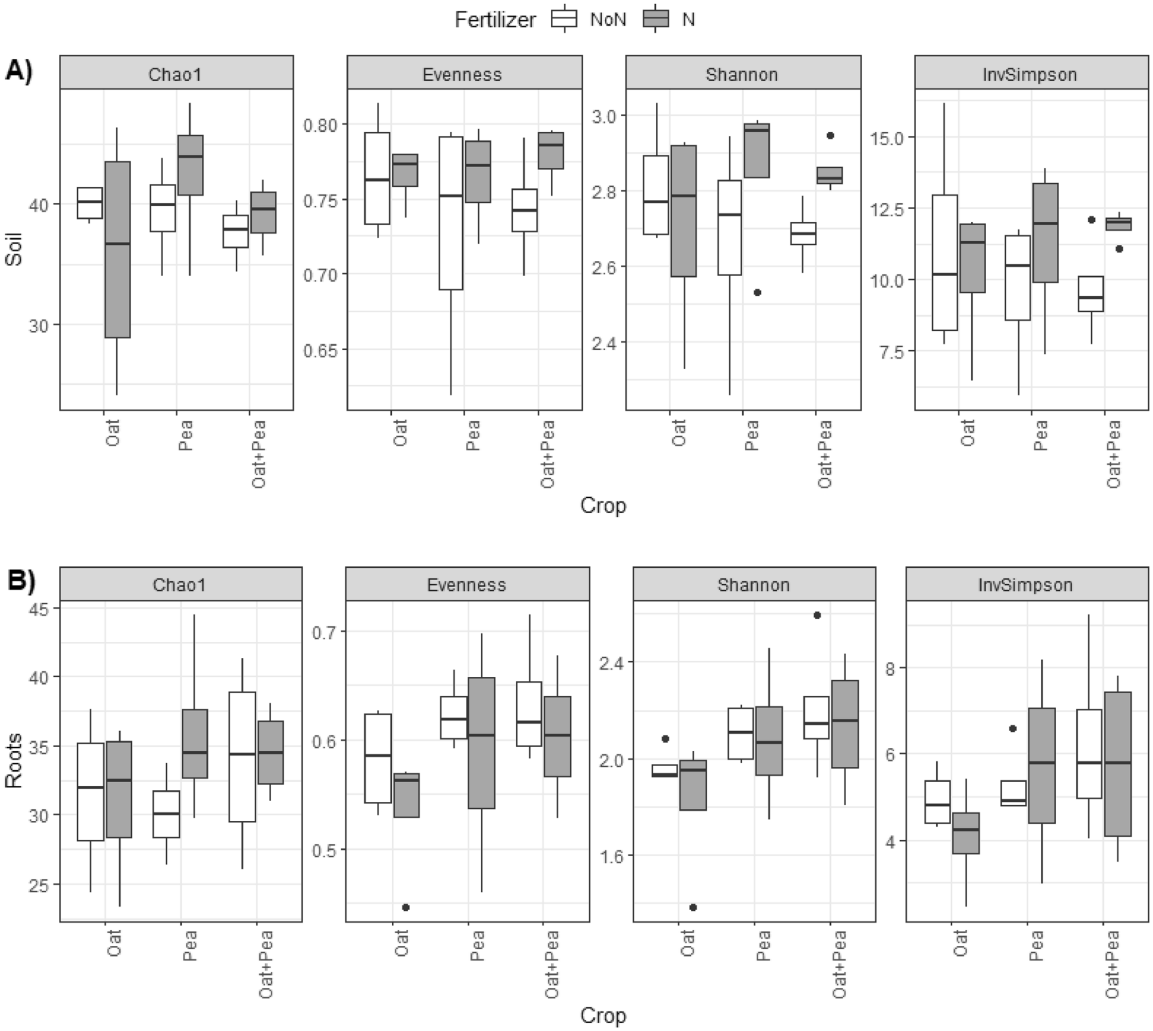
Arbuscular mycorrhizal fungi (AMF) alpha diversity including Chao1 richness, Peilou’s Evenness, Shannon diversity and inverse Simpson diversity across cropping systems at (**A**) bulk soil and (**B**) roots in 2020.

We found differences in AMF species richness estimates in the roots across treatment combinations (i.e., intercropping systems × N fertilizer rate) in 2019 (Chao1, p < 0.05, Supplementary Table 1). In oat, a decrease in AMF richness occurred in soils receiving N fertilizer, while an increase in AMF richness occurred in pea with the N fertilizer application (Fig. 1B, Chao1). The richness of AMF species remained relatively similar in oat-pea irrespective of N fertilizer addition (Fig. 1B, Chao1), and there were no differences in alpha diversity in bulk soils (Fig. 1A, Supplementary Table 1). No significant differences in species richness were observed in 2020 (Fig. 2A, Supplementary Table 1). Instead, we observed a difference in AMF species diversity in the roots across the cropping systems, a pattern not evident in 2019 (Shannon, Supplementary Table 1). Pea and oat-pea had the highest AMF diversity, while oat had the lowest (Fig. 2B, Shannon).

### Community composition

**Figure 3:**
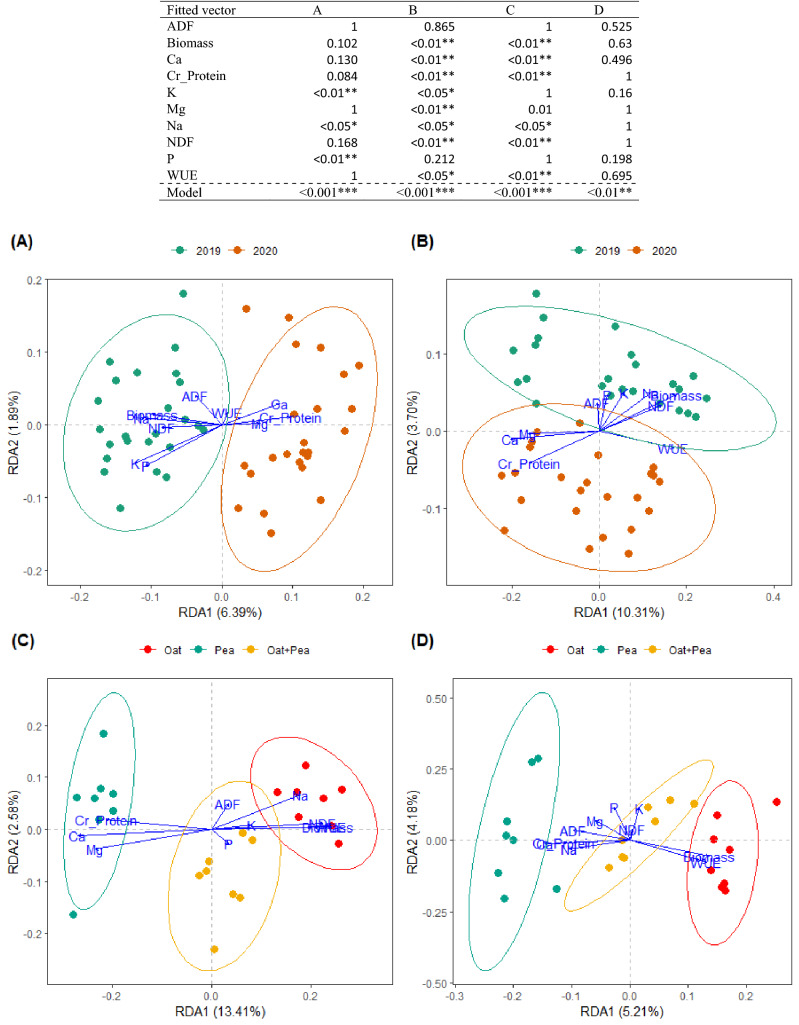
RDA of AMF taxonomic community composition among soil (**A**) and root (**B**) samples for two different years. RDA of AMF taxonomic community compositions across cropping treatments [Oat, Pea, Oat-Pea] in 2019 (**C**) and 2020 (**D**).

Differences in AMF community composition between the two study years in the bulk soil were significantly associated with forage nutritive indicators across all experimental units (Fig. 3A), specifically K, Na, and P concentrations in harvested forages (Ps < 0.05, model P < 0.001). In the root compartment, the AMF community composition between the two study years were also significantly associated with forage nutritive indicators (Fig. 3B), specifically neutral detergent fibre (NDF), calcium (Ca), potassium (K), magnesium (Mg), and sodium (Na) concentrations, biomass productivity as well as WUE (Ps < 0.05, model P < 0.001). Differences in AMF community composition across cropping systems within 2019 were significantly associated with forage indicators (Fig. 3C), specifically biomass, crude protein, NDF, WUE, and most mineral contents (Ca, K, Mg, Na) in the harvested forage (Ps < 0.05, model P < 0.001). In 2020, while the differences in AMF community composition between cropping systems were significantly associated with forage nutritive indicators (model P < 0.01), the fitted vectors did not significantly correlate with any of the AMF community composition of each cropping system (Fig. 3D).

**Figure 4:**
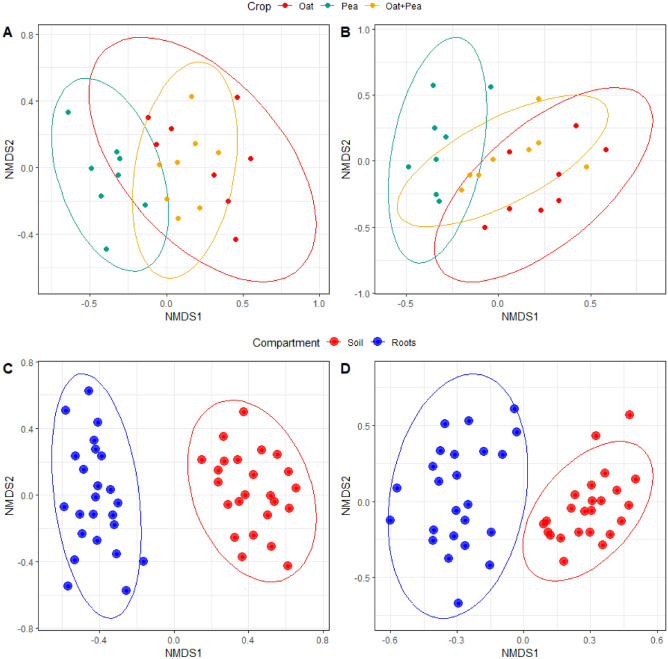
Non-metric multidimensional scaling (NMDS) plots of AMF communities based on Bray–Curtis distances. Circles are 95% confidence ellipses of the comparison, roots community composition significantly differs between: cropping systems in (**A**) 2019 and (**B**) 2020, compartments (bulk soil vs. roots) in (**C**) 2019 and (**D**) 2020.

NMDS plots showed spread in root AMF community composition between cropping options (Fig. 4A,B), which were found to be significant using PERMANOVA. Furthermore, the pairwise community composition comparisons indicated that the AMF communities were significantly different across each of the three cropping systems (*Ps* < 0.05; Table 2, Supplementary Table 2).

**Table 2:** Pairwise community composition comparison between cropping options in root samples based on PERMANOVA. p value adjusted by false discovery rate (FDR).

	Oat	Pea
2019-Roots (F = 15.27, p < 0.001***)		
Pea	0.0015	–
Oat-Pea	0.0020	0.0015
2020-Roots (F = 7.01, p < 0.001***)		
Pea	0.003	
Oat-Pea	0.005	0.003

Significance codes: *p* < 0.05, *; *p* < 0.01, **; *p* < 0.001, ***.

Root and soil AMF communities were also different (Supplementary Table 2). AMF alpha diversity between the two compartments was significantly different in both years (Supplementary Table 3). Not only was there a difference in richness of AMF species (Chao1, P < 0.05, Supplementary Table 3), but there was also a difference in species diversity (Evenness, Shannon, InvSimpson, *Ps* < 0.05, Supplementary Table 3). NMDS plots indicated no overlap in AMF community between the roots and soil compartment (Fig. 4C,D) with PERMANOVA confirming a significant difference between two compartments (Supplementary Table 2). Noticeably, this effect was observed when we pooled the AMF communities across all experimental samples; the similarities were greater between compartments (roots vs. bulk soil) rather than between years (2019 vs. 2020) (Supplementary Fig. 1).

While the soil community alpha diversity did not differ significantly (Supplementary Table 1), we did observe overlapped community composition when comparing between intercropping systems (Supplementary Fig. 2A,B), as well as in N fertilizer rates (Supplementary Fig. 2C–F).

**Figure 5:**
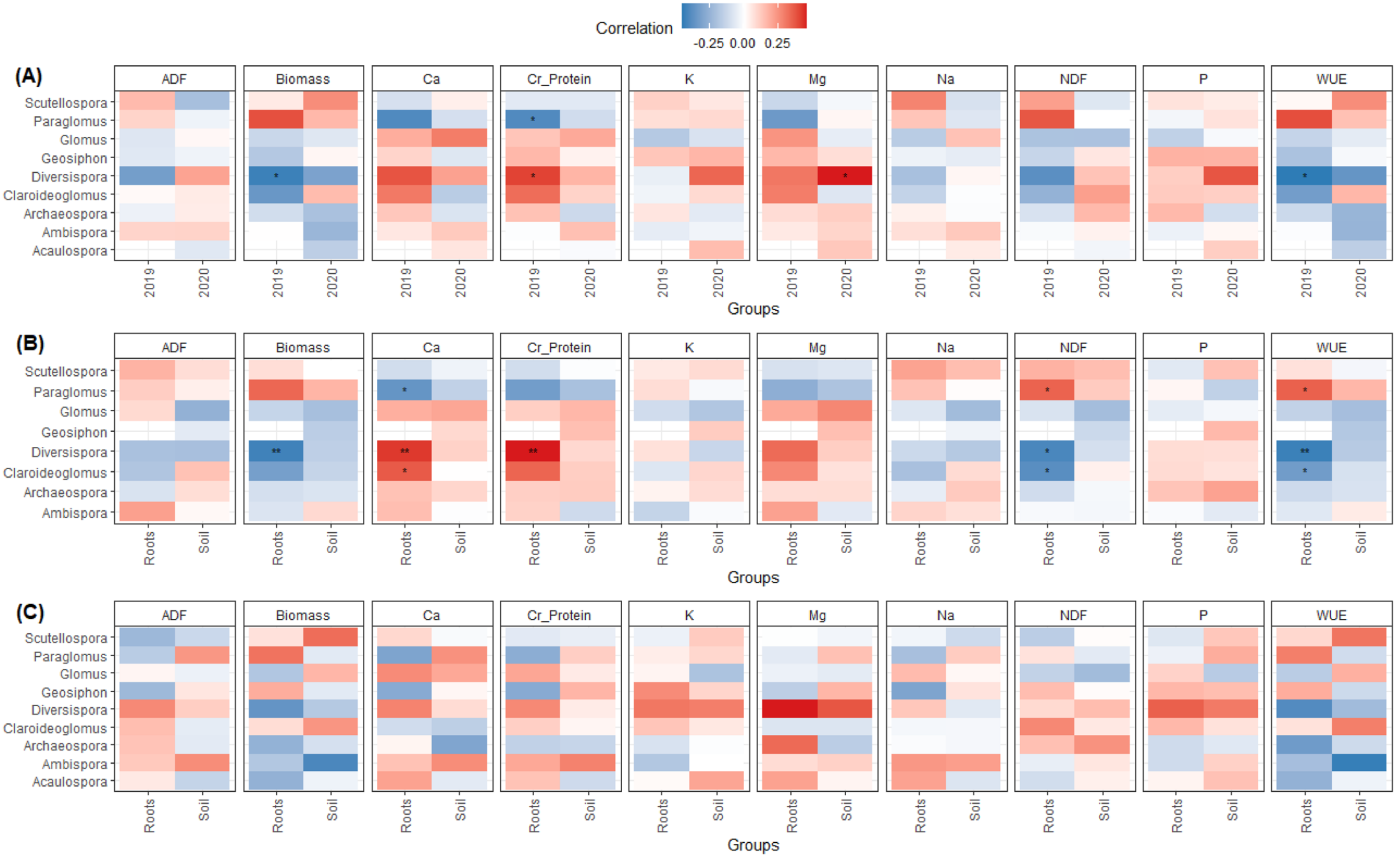
The heatmap of the correlation between AMF genus rank and physiochemical characteristics of (**A**) 2 years (2019 and 2020) and (**B**) compartments in 2019 and (**C**) compartments in 2020. Spearman’s correlation analysis was used for creating the heatmap. Positive correlation is shown in red, whilst negative correlation is shown in blue. The asterisks mean significant correlations (*p < 0.05; **p < 0.01).

*Diversispora* abundance in 2019 was negatively correlated with both biomass production and WUE, while positively correlated to crude protein content in the forage (Ps < 0.05, Fig. 5A). Conversely, crude protein was negatively correlated with *Paraglomus* in 2019, compared to *Diversispora* (P < 0.05). In 2020, *Diversispora* abundance was positively correlated with forage Mg concentration (P < 0.05, Fig. 5A). There were significant correlations with specific mycorrhizal genera in the roots in 2019 (Ps < 0.05, Fig. 5B); *Diversispora* abundance was negatively correlated with forage biomass production while positively correlated with forage Ca and crude protein contents (Ps < 0.01), and *Paraglomus* abundance was negatively correlated with Ca while *Claroideoglomus* abundance was positively correlated with Ca (Ps < 0.05). In the same year, NDF and WUE were positively correlated with *Paraglomus* abundance, but negatively correlated with *Diversispora* and *Claroideoglomus* (Ps < 0.05, Fig. 5B). No significant correlations in both soil and root compartments could be found in 2020 (Fig. 5C).

**Figure 6:**
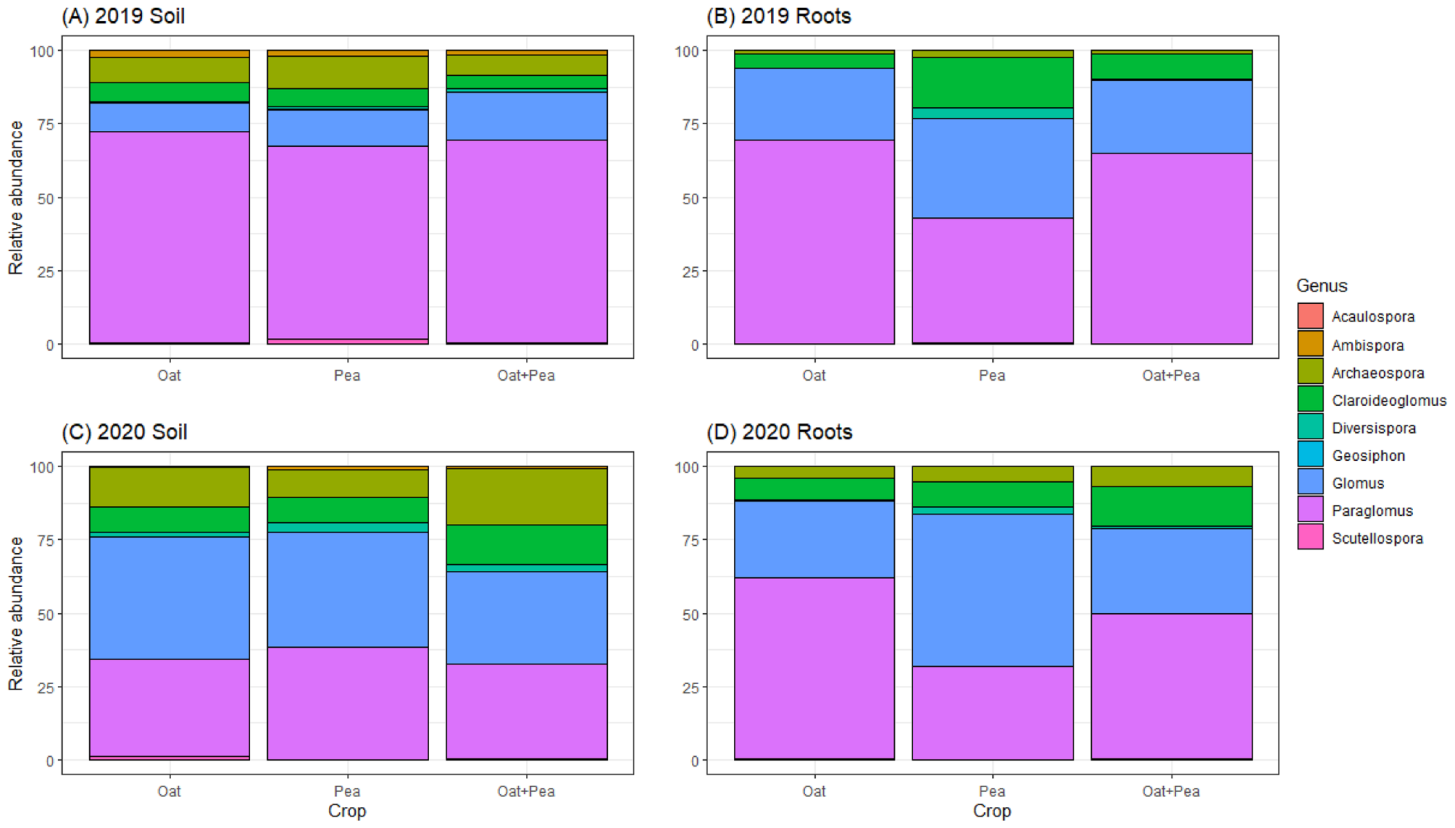
Proportional composition of AMF root and soil communities. Panel indicates proportional sequence abundance at site. Legend shows phylogenetic level to identified AMF genus.

In terms of relative abundance, 80.14% of the AMF community was assigned to phylum Glomeromycota in 2019, and 67.74% in 2020 (Fig. 6; Supplementary Fig. 3). Differential abundance analysis was implemented to see how cropping systems could influence specific genera. There were significant treatment impacts observed within the soil and root compartments (*Ps* < 0.05; Supplementary Tables 4, 5, 6, 7), as well as significant differences when comparing the bulk soil vs. root compartments (*Ps* < 0.05; Supplementary Table 8; Fig. 6). However, there was no significant effects of N fertilizer addition on AMF community within the soil or root compartments (Supplementary Tables 4, 5, 6, 7).

In the roots compartment in 2019, a proportional difference was observed in genera *Claroideoglomus* and *Diversispora* between oat and pea (p < 0.01, Supplementary Table 7). There was significantly higher content of *Claroideoglomus* and *Diversispora* in pea compared to oat (Fig. 6B). In the same year, between pea and oat-pea, a proportional difference was observed in *Paraglomus* (Supplementary Table 5). There was significantly higher content of *Paraglomus* in oat-pea compared to pea (Fig. 6B). In the soil compartment in 2020, a proportional difference was observed in *Archaeospora* between pea and oat-pea (P < 0.01, Supplementary Table 5). There was a significant higher presence of *Archaeospora* in oat-pea compared to pea alone (Fig. 6C). In the roots compartment in 2020, *Diversispora* abundance was higher in pea compared to oat, while *Paraglomus* abundance was highest in oat (*Ps* < 0.05; Supplementary Table 7, Fig. 6D). In terms of compartmental differences (roots vs. bulk soil), *Ambispora* and *Archaeospora* were found to be significantly different in both years, *Claroideoglomus* and *Glomus* in 2019, and *Paraglomus* in 2020 (Supplementary Table 8). In both years, there was a higher abundance of *Acaulospora* and *Archaeospora* in the bulk soil compartment compared to the roots compartment (*Ps* < 0.05; Supplementary Table 8, Supplementary Fig. 4). There was a higher abundance of *Claroideoglomus* and *Glomus* in the root compartment in 2019 (*Ps* < 0.05; Supplementary Table 8, Supplementary Fig. 4A), and there was a higher abundance of *Paraglomus* in the root compartment in 2020 (*P* < 0.05; Supplementary Table 8, Supplementary Fig. 4B).

### Root colonization

In the counting of hyphae, arbuscule, and vesicle infection in roots, we did not observe any interactions between cropping systems and N fertilizer addition (data not shown). Likewise, there were no significant effects of cropping systems or N fertilizer addition.

## Discussion

In this study, we found that field management factors including cropping choice and N fertilizer application had significant impacts on AMF diversity. We also show that, as previously demonstrated^34,35^, root and soil compartments host different AMF community composition. Previous studies have also shown that field management impacts AMF communities^34,36^.

We observed higher alpha diversity index values (Chao1 in 2019, Fig. 1A; Shannon in 2020, Fig. 2B) in oat-pea intercropping than in either monocropped oat or pea. Since the diversity increase in intercropping oat with pea does not equal the sum of the diversity of oat alone and pea alone, we infer that the mycorrhizal communities overlap between oat and pea, as well as synergy supporting a higher diversity of AMF when two functionally complimentary plant species are grown together. In combining the two crop species, the root exudates may help to recruit and maintain an environment suitable for mycorrhizal species commonly present in both plant species^36^.

In 2019, where the rainfall was below normal average (Table 1), AMF richness was affected by both cropping choice and N fertilizer addition (Supplementary Table 1). With oat and oat-pea, a decrease in AMF richness occurred when adding N fertilizer into the system. Conversely, pea experienced the exact opposite effect whereby adding N fertilizer increased AMF richness. In previous studies, N fertilization altered mycorrhizal communities^15,18^. Johnson^18^ suggested that the addition of N fertilizer causes plants to select for inferior species of mycorrhizae. With an increase in forage biomass production as a response to fertilizer addition in oat^3^, there was a decrease in AMF richness (Fig. 1B). With this, one hypothesis is that oat, like perennial grasses, benefit from the N fertilizer addition, and thus would benefit less from mycorrhizal symbiosis^37^. This would lead to less recruitment, as mycorrhizal infection would deem to be an inefficient growing strategy. Since peas do not benefit from adding N fertilizer as this reduces rhizobium activity^38,39^, the recruitment of mycorrhiza becomes the more effective strategy for nutrient acquisition in pea fields receiving N fertilizer additions. As a result, intercropping oat and pea can be conceptualized as an effective strategy in managing the mycorrhizal community as oat and pea have different requirements of N fertilizer additions, which was reflected in how the relative abundance of mycorrhizal species remained similar when comparing with and without N fertilizer addition (Fig. 1A).

From observing differences between the root and soil samples, as well as between the treatments, we conclude that plant preferences determine the root mycorrhizal community. The soil mycorrhizal community, which was generally more diverse than the root community (Supplementary Table 3), serves as the inoculum pool that living roots select from and represents the spores deposited by plants historically present^36^. Studies have shown that monocropping, fungicide use, and synthetic nutrient application can reduce the inoculum pool, which limits the optimization of the mycorrhizal synergy^18,40,41^. Lack of plant diversity in monocropping decreases introduced spores into the system, and with reduced plant-fungal symbiosis due to synthetic nutrient dependency, the mycorrhizae are less likely to survive. Glyphosate application also impacts mycorrhizal symbiosis^42–44^ and was applied to the entire experiment as a treatment for pre-emergent weeds. While this is standard practice in northern grain cropping systems, and was uniformly applied to all treatments, it has been demonstrated that glyphosate negatively impacts AMF colonization of roots^42,43^ and AMF biomass in soil^44^. Glyphosate is degraded by soil organisms^45,46^, however, it can accumulate in northern soils where cold and freezing conditions slow degradation^47^. The legacy lack of plant diversity at this site, and the management history including nutrient use and glyphosate application likely reduced the AMF we detected and described in this study.

Within the observed variety of AMF genera in the roots, *Diversispora* was significantly more abundant in pea than in oats in each of the two growing seasons. Additionally, while no significant difference was found between oat and oat-pea, oat-pea does consistently have higher abundance of *Diversispora* than oat (Supplementary Tables 6, 7). Curiously, *Clarideoglomus* was greatly recruited only by peas in 2019 under drier soil conditions (Fig. 6B), and not in 2020 (Fig. 6D). This phenomenon of peas exclusively recruiting *Clarideoglomus* was seen in both the bulk soil and roots compartments (Supplementary Fig. 4). Collectively, this finding can suggest that this AMF genus could be moisture sensitive. We thus hypothesize that the genus *Diversispora* is a key group of mycorrhizae that peas depend on more consistently, while that the genus *Clarideoglomus* is facultatively recruited depending upon the prevalence of dry climate conditions. However, there lacks research detailing the roles and functions of this genus, hence this is a topic that requires further exploration.

Similarly, we witnessed that *Paraglomus* was significantly higher in oat-pea than in pea in the drier 2019, while significantly lower in oat-pea than in oat in the wetter 2020. This relationship could reveal which plant species within the intercropping system the mycorrhizal community is prioritizing to associate with as a function of underlying soil moisture conditions. This is inferred because the *Paraglomus* abundance in oat-pea was between oat and pea; however, it was more similar to one of the two monocrops depending on experiencing high or low rainfall during a given growing season. As peas can access deeper water reservoirs due to their taproot system, they would tend to grow better in drier environments compared to oats, which would have a shallower fibrous root system. Conversely, a taproot system would not be able to advantageously capitalize on moisture resources in a wetter environment. To elaborate further, this finding may be an indication that pea exudation naturally deters the presence of *Paraglomus*, which could imply that this AMF genus might be harmful or not beneficial for pea growth. As plant stress is a factor that increases exudation^48,49^, this result demonstrates that within an intercropped field, the stressed plants can persist by accessing soil resources via the fungal network, while the dominant plant species without stress simply capitalizes directly on the favorable conditions. While this finding does not refute the notion that mycorrhizae have a transport channel that shares nutrients among plants (both intra-species and inter-species)^13,14^, it does pose the question as to whether mycorrhizae simply share the nutrients gathered from the soil solution through the fungal network rather than transferring nutrients directly from one plant to another within a field.

## Conclusion

This study enforces that AMF communities are highly controlled by plant–soil interactions, specifically how different plant species and growing conditions play a role in the structuring of mycorrhizal communities. This study also demonstrated that mycorrhizal species richness increases with intercropping likely because root exudation from multiple plant species in intercropping can recruit mycorrhizae and create an overlapped AMF community that collectively benefits the intercropped plant species. With N fertilization, we noticed a decrease in richness in AMF species. Furthermore, we ascertained two AMF genera indicators: (i) *Diversispora* presence indicated good growing environments for peas, and (ii) *Paraglomus* was linked to a stressed plant species within the oat-pea intercropping system.

Overall, this study prompts further research to focus on the optimal N fertilizer addition to balance between adequate yield gain and AMF species retention in the soil, as well as the role of *Diversispora* and *Paraglomus* in oat and peas monocrops and intercropping. Specifically, why these AMF genera need to be present or absent to underpin the growth of annual crop species planted alone or in combination.

## Supplementary Information

Supplementary Information.

## Data Availability

The dataset generated during the current study are available from PRJEB53715 at the European Nucleotide Archive. https://www.ebi.ac.uk/ena/browser/view/PRJEB53715.

## Abbreviations

AMF: Arbuscular mycorrhizal fungi
Ca: Calcium
K: Potassium
Mg: Magnesium
Na: Sodium
N: Nitrogen
NDF: Neutral detergent fiber
WUE: Water use efficiency
