# Supplementary material for: Arbuscular mycorrhizal fungi in oat-pea intercropping
Source: Sci Rep. 2023 Jan 9;13:390. doi: 10.1038/s41598-022-22743-7 (PMC9829737; doi:10.1038/s41598-022-22743-7)
Supplement: Supplementary file 1 — Supplementary Information. [file 41598_2022_22743_MOESM1_ESM.docx]

**Supplementary figures and tables for**

**Arbuscular mycorrhizal fungi in oat-pea intercropping**

Alan Lee^a,c^, Patrick Neuberger^b^, Akim Omokanye^a,c^, Guillermo Hernandez-Ramirez^c^, Keunbae Kim^c^, and Monika A. Gorzelak^b^

^a,b^Peace Country Beef and Forage Association, Fairview, AB T0H1L0, Canada. akim@pcbfa.ca

^b^ Agriculture and Agri-Food Canada, Lethbridge Development and Research Centre, 5403-1 Avenue South, Lethbridge, AB T1J 4B1, Canada

^c^Department of Renewable Resources, University of Alberta, Edmonton, AB T6G 2R3, Canada. [alan6@ualberta.ca](mailto:alan6@ualberta.ca), [ghernand@ualberta.ca](mailto:ghernand@ualberta.ca), keunbae@ualberta.ca

**Corresponding Author:** Monika A. Gorzelak monika.gorzelak@agr.gc.ca

**Supplementary Table 1.** Alpha diversity in bulk soil and roots samples across the crop × N fertilizer effects with two-way ANOVA in 2019 and 2020.

|  |  | Soil |  | Roots |  |
| --- | --- | --- | --- | --- | --- |
|  |  | 2019 |  |  |  |
| Parameter | Treatment | F-value | P-value | F-value | P-value |
| Chao1 | Crop | 0.1277 | 0.8809 | 1.5972 | 0.2350 |
|  | Fertilizer | 0.0286 | 0.8677 | 0.0000 | 1.0000 |
|  | Crop: Fertilizer | 1.8561 | 0.1904 | 6.2173 | <0.05* |
| Evenness | Crop | 0.127 | 0.8809 | 1.1698 | 0.3342 |
|  | Fertilizer | 0.0286 | 0.8677 | 0.1282 | 0.7247 |
|  | Crop: Fertilizer | 0.6214 | 0.5505 | 0.5653 | 0.5799 |
| Shannon | Crop | 0.1277 | 0.8809 | 1.7151 | 0.2097 |
|  | Fertilizer | 0.0286 | 0.8677 | 0.1706 | 0.6847 |
|  | Crop: Fertilizer | 0.1875 | 0.8310 | 1.8105 | 0.1975 |
| InvSimpson | Crop | 0.1277 | 0.8809 | 1.3639 | 0.2822 |
|  | Fertilizer | 0.0286 | 0.8677 | 0.2454 | 0.6267 |
|  | Crop: Fertilizer | 0.1460 | 0.8653 | 1.9728 | 0.1735 |
|  |  | 2020 |  |  |  |
| Chao1 | Crop | 0.7225 | 0.4999 | 0.6277 | 0.5457 |
|  | Fertilizer | 0.0069 | 0.9348 | 0.8229 | 0.3770 |
|  | Crop: Fertilizer | 0.9727 | 0.4007 | 0.7605 | 0.4846 |
| Evenness | Crop | 0.400 | 0.6762 | 2.9941 | 0.0769 |
|  | Fertilizer | 1.839 | 0.1928 | 2.9000 | 0.1068 |
|  | Crop: Fertilizer | 0.432 | 0.6573 | 0.0587 | 0.9432 |
| Shannon | Crop | 0.0366 | 0.9641 | 4.9846 | <0.05* |
|  | Fertilizer | 1.3474 | 0.2618 | 1.0715 | 0.3151 |
|  | Crop: Fertilizer | 1.264 | 0.3110 | 0.2116 | 0.8166 |
| InvSimpson | Crop | 0.0218 | 0.9785 | 2.90686 | 0.0821 |
|  | Fertilizer | 0.9228 | 0.3502 | 0.40540 | 0.5328 |
|  | Crop: Fertilizer | 0.7504 | 0.4891 | 0.50203 | 0.6151 |

Number of degree freedom (NumDF) is [Crop = 2, Fertilizer = 1, Crop: Fertilizer = 2].

Denominator of Degree of freedom (denDF) is 15 and 17, in soil and roots, respectively.

Supplementary **Table 2.** PERMANOVA across combination cropping systems (crop × N fertilizer) and root compartments (bulk soil vs. roots).

|  |  |  | 2019 |  |  | 2020 |  |
| --- | --- | --- | --- | --- | --- | --- | --- |
| Treatment |  | F.model | R^2^ | Pr(>F) | F.model | R^2^ | Pr(>F) |
|  |  |  |  | Bulk soil |  |  |  |
| Crop |  | 0.728 | 0.067 | 0.743 | 1.012 | 0.092 | 0.276 |
| Fertilizer |  | 0.658 | 0.030 | 0.762 | 0.640 | 0.029 | 0.802 |
| Crop : Fertilizer |  | 0.810 | 0.074 | 0.639 | 0.635 | 0.0579 | 0.923 |
|  |  |  |  | Roots |  |  |  |
| Crop |  | 14.92 | 0.592 | <0.001*** | 6.483 | 0.400 | <0.001*** |
| Fertilizer |  | 0.254 | 0.005 | 0.906 | 0.524 | 0.016 | 0.698 |
| Crop : Fertilizer |  | 1.134 | 0.045 | 0.379 | 0.451 | 0.027 | 0.878 |
|  |  |  |  | Root |  |  |  |
| Compartment |  | 39.179 | 0.460 | <0.001*** | 16.241 | 0.261 | <0.001*** |

Significance codes: *P*<0.05, *; *P*<0.01, **; *P*<0.001, ***.

Supplementary **Table 3.** AMF alpha diversity. Average and standard errors (n=24) of soil and root samples from 2019 and 2020 across treatments. Metrics include Chao1 richness, Peilou’s evenness, Shannon’s diversity, and inverse Simpson’s diversity. Italics indicate the P-value.

| Treatment | Chao1 | Evenness | Shannon | InvSimpson |
| --- | --- | --- | --- | --- |
|  | 2019 |  |  |  |
| Bulk soil | 24.12±1.08a¶ | 0.67±0.02b | 2.10±0.06b | 5.49±0.38b |
| Roots | 26.81±0.86b | 0.56±0.02a | 1.83±0.05a | 4.42±0.28a |
|  | *<0.05** | *<0.001**** | *<0.001**** | *<0.01*** |
|  | 2020 |  |  |  |
| Bulk soil | 39.10±1.07b | 0.75±0.01b | 2.76±0.04b | 10.62±0.50b |
| Roots | 32.80±1.06a | 0.59±0.01a | 2.05±0.05a | 5.31. ±0.34a |
|  | *<0.001**** | *<0.001**** | *<0.001**** | *<0.001**** |

¶Pairwise comparisons were performed with Tukey’s HSD test after ANOVA.

Lowercase letters signify significant difference between treatments based on Tukey HSD test after ANOVA

Supplementary **Table 4.** Differential abundance in genus rank impacted by treatments (crop × N fertilizer) in soil compartment in 2019.

| Genus | logFC | logCPM | LR | P-value | FDR |
| --- | --- | --- | --- | --- | --- |
|  | *Crop* |  |  |  |  |
|  | *Oat vs. Pea* |  |  |  |  |
| Acaulospora | 0.0000 | 6.6919 | 0.0000 | 1.0000 | 1.0000 |
| Ambispora | -0.1827 | 14.4043 | 0.0593 | 0.8076 | 1.0000 |
| Archaeospora | 0.3378 | 16.6234 | 0.5555 | 0.4561 | 1.0000 |
| Claroideoglomus | -0.1858 | 16.0487 | 0.0503 | 0.8225 | 1.0000 |
| Diversispora | 0.3296 | 12.8606 | 0.0267 | 0.8703 | 1.0000 |
| Geosiphon | 6.7054 | 9.1622 | 0.7148 | 0.3979 | 1.0000 |
| Glomus | 0.3810 | 16.7911 | 0.9856 | 0.3208 | 1.0000 |
| Paraglomus | -0.1210 | 19.3964 | 0.8122 | 0.3675 | 1.0000 |
| Scutellospora | 0.2404 | 12.7747 | 0.0051 | 0.9432 | 1.0000 |
|  | *Oat vs. Oat-Pea* |  |  |  |  |
| Acaulospora | 0.0000 | 6.9199 | 0.0000 | 1.0000 | 1.0000 |
| Ambispora | -0.4891 | 14.2243 | 0.4162 | 0.5189 | 0.7542 |
| Archaeospora | -0.2405 | 16.2658 | 0.3292 | 0.5661 | 0.7542 |
| Claroideoglomus | -0.4853 | 15.7479 | 0.4994 | 0.4798 | 0.7542 |
| Diversispora | 1.1069 | 13.2736 | 0.4184 | 0.5177 | 0.7542 |
| Geosiphon | 0.0000 | 6.9199 | 0.0000 | 1.0000 | 1.0000 |
| Glomus | 0.8955 | 17.0283 | 7.5819 | 0.0059 | 0.0531 |
| Paraglomus | 0.1025 | 19.4419 | 0.5779 | 0.4471 | 0.7542 |
| Scutellospora | -2.3916 | 11.2163 | 0.2957 | 0.5866 | 0.7542 |
|  | *Pea vs. Oat-Pea* |  |  |  |  |
| Acaulospora | 0.0000 | 6.6198 | 0.0000 | 1.0000 | 1.0000 |
| Ambispora | -0.5033 | 14.1489 | 0.5275 | 0.4676 | 0.7173 |
| Archaeospora | -0.4822 | 16.3873 | 2.5040 | 0.1136 | 0.5110 |
| Claroideoglomus | -0.2404 | 15.6921 | 0.2484 | 0.6182 | 0.7782 |
| Diversispora | 0.5859 | 13.3669 | 0.1573 | 0.6917 | 0.7782 |
| Geosiphon | -6.4775 | 8.9165 | 0.6698 | 0.4131 | 0.7173 |
| Glomus | 0.4702 | 17.1270 | 2.8194 | 0.0931 | 0.5110 |
| Paraglomus | 0.1491 | 19.4316 | 0.5030 | 0.4782 | 0.7173 |
| Scutellospora | -2.6625 | 13.1324 | 1.8339 | 0.1757 | 0.5270 |
|  | *N Fertilizer* |  |  |  |  |
| Acaulospora | 0.0000 | 6.7347 | 0.0000 | 1.0000 | 1.0000 |
| Ambispora | 0.5864 | 14.2646 | 1.1714 | 0.2791 | 0.5243 |
| Archaeospora | -0.0989 | 16.4048 | 0.1031 | 0.7482 | 0.9094 |
| Claroideoglomus | -1.0752 | 15.8327 | 4.2421 | 0.0394 | 0.1774 |
| Diversispora | 1.4918 | 13.2102 | 1.1136 | 0.2913 | 0.5243 |
| Geosiphon | 5.9028 | 8.5833 | 0.6269 | 0.4285 | 0.6427 |
| Glomus | 0.0711 | 17.0027 | 0.0588 | 0.8084 | 0.9094 |
| Paraglomus | -0.1983 | 19.4435 | 1.4910 | 0.2221 | 0.5243 |
| Scutellospora | 4.4595 | 12.8212 | 5.0883 | 0.0241 | 0.1774 |

Note: LogFC designates log fold change; LogCPM, log counts per million; LR, likelihood ratio; FDR, false discovery rate.

*, **, and *** show statistical significance at *P* <0.05, *P* <0.01, *P* <0.001, respectively.

Supplementary **Table 5.** Differential abundance in genus rank impacted by treatments (crop × N fertilizer × compartment) in soil compartment in 2020.

| Genus | logFC | logCPM | LR | P-value | FDR |
| --- | --- | --- | --- | --- | --- |
|  | *Crop* |  |  |  |  |
|  | *Oat vs. Pea* |  |  |  |  |
| Acaulospora | -0.8035 | 7.9600 | 0.0243 | 0.8762 | 0.8762 |
| Ambispora | 1.3911 | 12.7442 | 1.9456 | 0.1631 | 0.3669 |
| Archaeospora | -0.4631 | 16.8018 | 2.3644 | 0.1241 | 0.3669 |
| Claroideoglomus | -0.0751 | 16.4194 | 0.0321 | 0.8577 | 0.8762 |
| Diversispora | 1.0663 | 14.6686 | 2.2826 | 0.1308 | 0.3669 |
| Geosiphon | -1.2063 | 8.8371 | 0.0833 | 0.7728 | 0.8762 |
| Glomus | -0.2243 | 18.6496 | 1.1247 | 0.2889 | 0.4334 |
| Paraglomus | 0.2100 | 18.4620 | 1.2367 | 0.2661 | 0.4334 |
| Scutellospora | -5.2554 | 12.6132 | 4.3738 | 0.0365 | 0.3285 |
|  | *Oat vs. Oat-Pea* |  |  |  |  |
| Acaulospora | -2.0251 | 7.8935 | 0.1519 | 0.6967 | 0.8405 |
| Ambispora | 1.1497 | 12.5062 | 0.7125 | 0.3986 | 0.7175 |
| Archaeospora | 0.6636 | 17.3084 | 4.1327 | 0.0421 | 0.3174 |
| Claroideoglomus | 0.8312 | 16.7974 | 3.2705 | 0.0705 | 0.3174 |
| Diversispora | 1.2925 | 14.4156 | 1.6542 | 0.1984 | 0.5188 |
| Geosiphon | 0.2545 | 9.7585 | 0.0069 | 0.9337 | 0.9337 |
| Glomus | -0.2533 | 18.4851 | 1.4373 | 0.2306 | 0.5188 |
| Paraglomus | 0.0762 | 18.3246 | 0.1040 | 0.7471 | 0.8405 |
| Scutellospora | -2.5044 | 13.1511 | 0.2928 | 0.5884 | 0.8405 |
|  | *Pea vs. Oat-Pea* |  |  |  |  |
| Acaulospora | -1.5681 | 7.3397 | 0.1979 | 0.6564 | 0.8440 |
| Ambispora | -0.3632 | 13.0798 | 0.2636 | 0.6077 | 0.8440 |
| Archaeospora | 1.0840 | 17.1833 | 13.7664 | 0.0002 | P<0.01** |
| Claroideoglomus | 0.7903 | 16.7608 | 5.0433 | 0.0247 | 0.1112 |
| Diversispora | 0.1498 | 14.8059 | 0.0423 | 0.8371 | 0.9295 |
| Geosiphon | 0.9734 | 9.5768 | 0.0078 | 0.9295 | 0.9295 |
| Glomus | -0.2126 | 18.4218 | 1.7160 | 0.1902 | 0.5706 |
| Paraglomus | -0.1168 | 18.4252 | 0.5477 | 0.4593 | 0.8440 |
| Scutellospora | 2.3645 | 11.5246 | 0.4666 | 0.4945 | 0.8440 |
|  | *N Fertilizer* |  |  |  |  |
| Acaulospora | -0.7035 | 7.7010 | 0.0242 | 0.8765 | 0.9273 |
| Ambispora | 0.3361 | 12.7365 | 0.2220 | 0.6376 | 0.8490 |
| Archaeospora | -0.2095 | 17.1126 | 0.5014 | 0.4789 | 0.8490 |
| Claroideoglomus | -0.3184 | 16.6616 | 0.8260 | 0.3634 | 0.8177 |
| Diversispora | -0.6853 | 14.5830 | 1.1800 | 0.2773 | 0.8177 |
| Geosiphon | 0.4407 | 9.3635 | 0.0083 | 0.9273 | 0.9273 |
| Glomus | -0.2528 | 18.5733 | 1.6210 | 0.2030 | 0.8177 |
| Paraglomus | -0.1462 | 18.3868 | 1.0832 | 0.2980 | 0.8177 |
| Scutellospora | 1.4579 | 12.4333 | 0.1931 | 0.6603 | 0.8490 |

Note: LogFC designates log fold change; LogCPM, log counts per million; LR, likelihood ratio; FDR, false discovery rate.

*, **, and *** show statistical significance at *P* <0.05, *P* <0.01, *P* <0.001, respectively.

Supplementary **Table 6.** Differential abundance in genus rank impacted by treatments (crop × N fertilizer) roots compartment in 2019.

| Genus | logFC | logCPM | LR | P-value | FDR |
| --- | --- | --- | --- | --- | --- |
|  | *Crop* |  |  |  |  |
|  | *Oat vs. Pea* |  |  |  |  |
| Acaulospora | 0.0000 | 5.4251 | 0.0000 | 1.0000 | 1.0000 |
| Ambispora | 0.4397 | 9.4917 | 0.0613 | 0.8044 | 1.0000 |
| Archaeospora | 1.4919 | 14.1683 | 3.4482 | 0.0633 | 0.1425 |
| Claroideoglomus | 1.9763 | 16.8508 | 11.0531 | 0.0009 | <0.01** |
| Diversispora | 4.2749 | 14.5509 | 10.4065 | 0.0013 | <0.01** |
| Geosiphon | 0.0000 | 5.4251 | 0.0000 | 1.0000 | 1.0000 |
| Glomus | 0.4968 | 18.1753 | 1.1880 | 0.2757 | 0.4963 |
| Paraglomus | -0.6351 | 19.1178 | 3.9704 | 0.0463 | 0.1389 |
| Scutellospora | -0.1931 | 10.9673 | 0.0065 | 0.9355 | 1.0000 |
|  | *Oat vs. Oat-Pea* |  |  |  |  |
| Acaulospora | 0.0000 | 5.5051 | 0.0000 | 1.0000 | 1.0000 |
| Ambispora | -0.1349 | 9.3792 | 0.0060 | 0.9382 | 1.0000 |
| Archaeospora | 0.1549 | 13.2765 | 0.0532 | 0.8176 | 1.0000 |
| Claroideoglomus | 0.7652 | 15.9601 | 2.5889 | 0.1076 | 0.4843 |
| Diversispora | 1.0324 | 11.7888 | 0.4610 | 0.4972 | 1.0000 |
| Geosiphon | 0.0000 | 5.5051 | 0.0000 | 1.0000 | 1.0000 |
| Glomus | 0.0422 | 17.9462 | 0.0084 | 0.9271 | 1.0000 |
| Paraglomus | -0.1901 | 19.4004 | 0.5745 | 0.4485 | 1.0000 |
| Scutellospora | -5.6064 | 8.8917 | 3.6738 | 0.0553 | 0.4843 |
|  | *Pea vs. Oat-Pea* |  |  |  |  |
| Acaulospora | 0.0000 | 5.3631 | 0.0000 | 1.0000 | 1.0000 |
| Ambispora | -0.4987 | 9.6272 | 0.0665 | 0.7965 | 1.0000 |
| Archaeospora | -0.8562 | 14.0865 | 1.2727 | 0.2593 | 0.3889 |
| Claroideoglomus | -0.7357 | 16.9400 | 2.4024 | 0.1212 | 0.2726 |
| Diversispora | -2.2442 | 14.4101 | 3.7358 | 0.0533 | 0.1728 |
| Geosiphon | 0.0000 | 5.3631 | 0.0000 | 1.0000 | 1.0000 |
| Glomus | -0.5461 | 18.1151 | 1.6018 | 0.2056 | 0.3702 |
| Paraglomus | 1.0818 | 19.2547 | 8.0149 | 0.0046 | <0.05* |
| Scutellospora | -5.0267 | 11.6952 | 3.6051 | 0.0576 | 0.1728 |
|  | *N Fertilizer* |  |  |  |  |
| Acaulospora | 0.0000 | 5.4648 | 0.0000 | 1.0000 | 1.0000 |
| Ambispora | -0.6643 | 9.4827 | 0.1700 | 0.6801 | 1.0000 |
| Archaeospora | 0.4050 | 14.0053 | 0.3659 | 0.5452 | 1.0000 |
| Claroideoglomus | -0.3611 | 16.7550 | 0.4797 | 0.4885 | 1.0000 |
| Diversispora | -1.4589 | 14.1711 | 1.5484 | 0.2134 | 1.0000 |
| Geosiphon | 0.0000 | 5.4648 | 0.0000 | 1.0000 | 1.0000 |
| Glomus | 0.1082 | 18.1213 | 0.0695 | 0.7920 | 1.0000 |
| Paraglomus | -0.2918 | 19.1779 | 1.3498 | 0.2453 | 1.0000 |
| Scutellospora | -0.0588 | 10.4827 | 0.0010 | 0.9742 | 1.0000 |

Note: LogFC designates log fold change; LogCPM, log counts per million; LR, likelihood ratio; FDR, false discovery rate.

*, **, and *** show statistical significance at *P* <0.05, *P* <0.01, *P* <0.001, respectively.

Supplementary **Table 7.** Differential abundance in genus rank impacted by treatments (crop × N fertilizer × compartment) in the roots compartment in 2020.

| Genus | logFC | logCPM | LR | P-value | FDR |
| --- | --- | --- | --- | --- | --- |
|  | *Crop* |  |  |  |  |
|  | *Oat vs. Pea* |  |  |  |  |
| Acaulospora | 1.3400 | 5.7791 | 0.4181 | 0.5179 | 0.7769 |
| Ambispora | 0.3758 | 8.8405 | 0.0480 | 0.8265 | 0.8265 |
| Archaeospora | 0.2148 | 15.4236 | 0.1393 | 0.7090 | 0.8265 |
| Claroideoglomus | 0.1373 | 16.3185 | 0.0664 | 0.7966 | 0.8265 |
| Diversispora | 4.9308 | 13.8508 | 14.1982 | 0.0002 | <0.001*** |
| Geosiphon | -2.9298 | 7.1943 | 1.1140 | 0.2912 | 0.5242 |
| Glomus | 0.9078 | 18.6071 | 5.7419 | 0.0166 | 0.0497 |
| Paraglomus | -0.9956 | 18.8468 | 15.3313 | 0.0001 | <0.001*** |
| Scutellospora | -4.1443 | 10.5788 | 1.7651 | 0.1840 | 0.4140 |
|  | *Oat vs. Oat-Pea* |  |  |  |  |
| Acaulospora | 0.0000 | 5.7105 | 0.0000 | 1.0000 | 1.0000 |
| Ambispora | 0.7853 | 9.1257 | 0.2531 | 0.6149 | 0.9731 |
| Archaeospora | 0.0805 | 15.7239 | 0.0187 | 0.8912 | 1.0000 |
| Claroideoglomus | 0.2639 | 16.5379 | 0.2075 | 0.6487 | 0.9731 |
| Diversispora | 2.9417 | 12.3120 | 2.2484 | 0.1338 | 0.6019 |
| Geosiphon | -2.9781 | 7.3468 | 1.0293 | 0.3103 | 0.9310 |
| Glomus | -0.3128 | 18.1497 | 0.4128 | 0.5206 | 0.9731 |
| Paraglomus | -0.9081 | 19.1818 | 9.3501 | 0.0022 | <0.05* |
| Scutellospora | -1.7704 | 11.4713 | 0.0845 | 0.7712 | 0.9916 |
|  | *Pea vs. Oat-Pea* |  |  |  |  |
| Acaulospora | -1.2608 | 5.7162 | 0.4546 | 0.5002 | 0.6431 |
| Ambispora | 0.8375 | 9.3984 | 0.3100 | 0.5777 | 0.6499 |
| Archaeospora | 0.4090 | 15.7980 | 0.5193 | 0.4712 | 0.6431 |
| Claroideoglomus | 0.7278 | 16.7840 | 1.5738 | 0.2097 | 0.4721 |
| Diversispora | -1.3748 | 14.0178 | 1.5728 | 0.2098 | 0.4721 |
| Geosiphon | 0.0000 | 5.5822 | 0.0000 | 1.0000 | 1.0000 |
| Glomus | -0.6857 | 18.7090 | 2.6929 | 0.1008 | 0.4536 |
| Paraglomus | 0.7613 | 18.6467 | 6.5095 | 0.0107 | 0.0966 |
| Scutellospora | 3.2193 | 10.5885 | 0.8959 | 0.3439 | 0.6190 |
|  | *N Fertilizer* |  |  |  |  |
| Acaulospora | 1.2060 | 5.7364 | 0.4907 | 0.4836 | 0.9652 |
| Ambispora | 0.3215 | 9.1175 | 0.0633 | 0.8013 | 0.9652 |
| Archaeospora | 0.1353 | 15.6430 | 0.0819 | 0.7747 | 0.9652 |
| Claroideoglomus | -0.0237 | 16.7823 | 0.0019 | 0.9652 | 0.9652 |
| Diversispora | -0.5084 | 13.6338 | 0.2227 | 0.6370 | 0.9652 |
| Geosiphon | -3.6210 | 6.8385 | 1.3155 | 0.2514 | 0.9652 |
| Glomus | -0.5601 | 18.4693 | 2.2882 | 0.1304 | 0.9652 |
| Paraglomus | -0.0612 | 18.8838 | 0.0533 | 0.8173 | 0.9652 |
| Scutellospora | -0.6344 | 11.0411 | 0.0105 | 0.9185 | 0.9652 |

Note: LogFC designates log fold change; LogCPM, log counts per million; LR, likelihood ratio; FDR, false discovery rate.

*, **, and *** show statistical significance at *P* <0.05, *P* <0.01, *P* <0.001, respectively.

Supplementary **Table 8.** Differential abundance in genus rank impacted by compartment (bulk soil vs. roots) in 2019 and 2020.

| Genus | logFC | logCPM | LR | P-value | FDR |
| --- | --- | --- | --- | --- | --- |
|  | *Compartment (bulk soil vs. roots)* | | | | |
|  | 2019 |  |  |  |  |
| Acaulospora | 0.0000 | 5.8542 | 0.0000 | 1.0000 | 1.0000 |
| Ambispora | -4.5508 | 13.2972 | 45.3874 | 0.0000 | <0.001*** |
| Archaeospora | -2.2978 | 15.5694 | 46.1072 | 0.0000 | <0.001*** |
| Claroideoglomus | 1.1599 | 16.2402 | 13.6099 | 0.0002 | <0.001*** |
| Diversispora | 0.7006 | 13.4025 | 0.6320 | 0.4266 | 0.5485 |
| Geosiphon | -5.4037 | 7.2650 | 1.1047 | 0.2932 | 0.5278 |
| Glomus | 1.5399 | 17.7457 | 35.8181 | 0.0000 | <0.001*** |
| Paraglomus | 0.1307 | 19.5759 | 0.2346 | 0.6282 | 0.7067 |
| Scutellospora | -1.5090 | 12.1991 | 0.8303 | 0.3622 | 0.5433 |
|  | 2020 |  |  |  |  |
| Acaulospora | -1.7607 | 6.7716 | 0.6778 | 0.4104 | 0.4617 |
| Ambispora | -3.4070 | 11.7094 | 33.1994 | 0.0000 | <0.001*** |
| Archaeospora | -0.9622 | 16.5093 | 12.2553 | 0.0005 | <0.01** |
| Claroideoglomus | 0.3499 | 16.6182 | 1.5912 | 0.2072 | 0.2663 |
| Diversispora | -0.7920 | 14.0620 | 1.6896 | 0.1936 | 0.2663 |
| Geosiphon | -3.2432 | 8.4337 | 3.3329 | 0.0679 | 0.1222 |
| Glomus | 0.3701 | 18.4968 | 3.6675 | 0.0555 | 0.1222 |
| Paraglomus | 1.0221 | 18.7609 | 38.7068 | 0.0000 | <0.001*** |
| Scutellospora | -1.1254 | 11.9039 | 0.3896 | 0.5325 | 0.5325 |

Note: LogFC designates log fold change; LogCPM, log counts per million; LR, likelihood ratio; FDR, false discovery rate.

*, **, and *** show statistical significance at *P* <0.05, *P* <0.01, *P* <0.001, respectively.


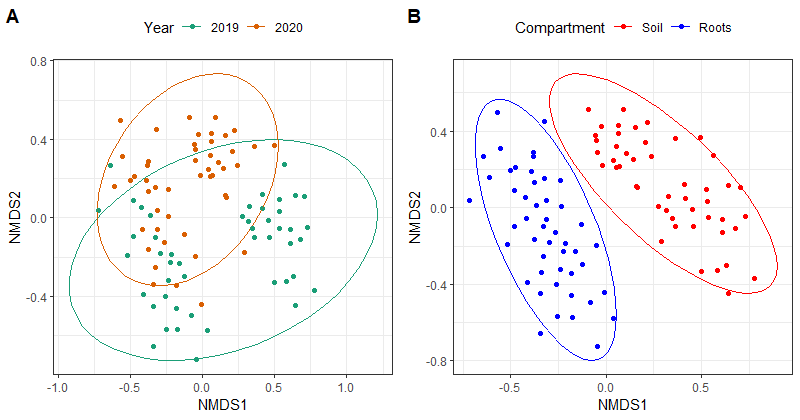


**Supplementary Fig. 1.** Non-metric multidimensional scaling (NMDS) plots of AMF communities based on Bray-Curtis distances. Circles are 95 % confidence ellipses of the comparison, community composition significantly differs between; (A) years and (B) compartment (bulk soil vs. roots).


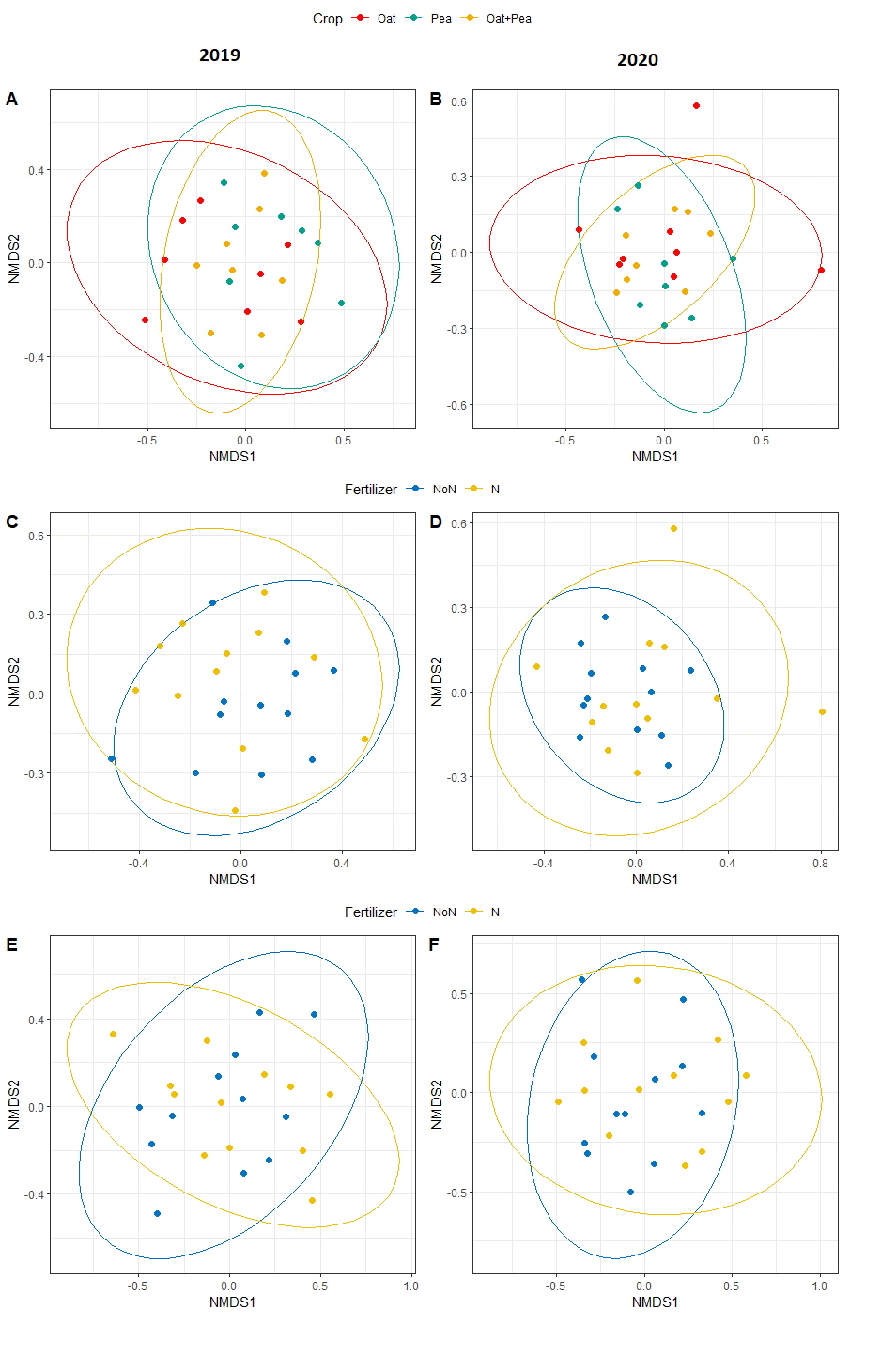


**Supplementary Fig. 2.** Non-metric multidimensional scaling (NMDS) plots of AMF communities based on Bray-Curtis distances. Circles are 95 % confidence ellipses of the comparison, soil community composition significantly differs between; cropping systems (A and B) and N fertilizer treatment (C, D, E and F). Left panels indicate 2019 data while right panels show 2020 data.


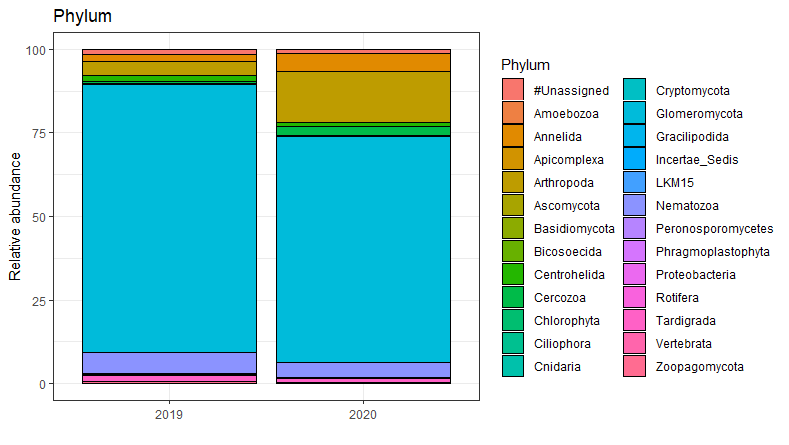


**Supplementary Fig. 3.** Proportional composition of AMF communities. The panel indicates proportional sequence abundance in each of the two study years. Legend shows phylogenetic level to identified AMF phylum.

2019: 80.14 % assigned to Glomeromycota, 1.50% not assigned to any Phylum

2020: 67.74 % assigned to Glomeromycota 1.23% not assigned to any Phylum


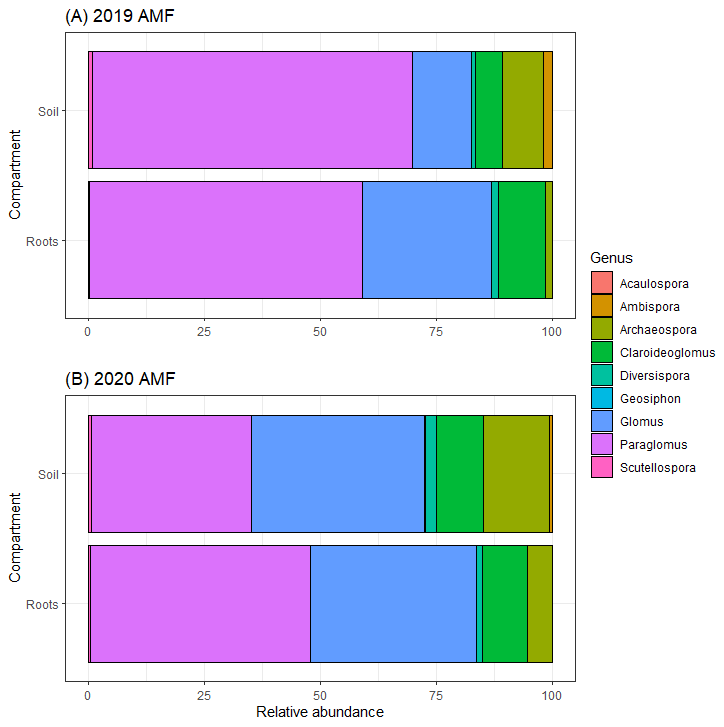


**Supplementary Fig. 4.** Proportional composition of AMF root and bulk soil communities in 2019 (A) and 2020 (B). Legend shows phylogenetic level to identified AMF genus.
